# Breaking Bad: how does a neuronal protein cause neuroendocrine cancer?

**DOI:** 10.18632/oncotarget.1710

**Published:** 2013-12-19

**Authors:** Karine Pozo, Fiemu E. Nwariaku, James A. Bibb

**Affiliations:** The University of Texas Southwestern Medical Center Dallas

Medullary thyroid carcinomas (MTC) are slow-growing tumors originating from neuroendocrine, parafollicular cells (C-cells) in the thyroid gland. Despite its low prevalence, MTC is one of the most deadly forms of thyroid cancers. Like other neuroendocrine cancers (e.g. pancreatic), MTC is often diagnosed at advanced stages at which surgical removal of the thyroid is no longer an effective treatment. MTC affects both men and women equally, is either sporadic (75% MTC cases) or hereditary (25% MTC cases) and associated to other neuroendocrine cancers (Multiple Endocrine Neoplasia syndrome, MEN2).

About 20 years ago, a breakthrough discovery revealed that activating germline mutations in the *Rearranged-during-transfection* (*RET*) protooncogene are responsible for 100% hereditary forms of the disease and about 40% sporadic forms of MTC [1, 2]. As a result, a large effort was set out to understand how *RET*, which encodes a receptor tyrosine kinase, causes MTC. Furthermore, molecular therapies targeting RET and other receptors tyrosine kinase have been developed which have led to the recent approval of two new drugs by the FDA. However these drugs have limited benefits and a large proportion of MTC patients still have no access to an efficient treatment because the causes of sporadic MTC are not well understood [3].

In our study [4], we show that the protein kinase, cyclin-dependent kinase 5 (Cdk5), plays a crucial role in sporadic MTC proliferation and demonstrate that blocking Cdk5 activity or its downstream effectors reduces significantly MTC proliferation. Cdk5 and its activators are present in normal human thyroid tissues and are enriched in hereditary and sporadic MTC patient specimen. Furthermore specific targeting of Cdk5 activity or Cdk5 expression arrests the proliferation of sporadic MTC cell lines that were derived from patient tumors. We corroborated this result *in vivo* in a transgenic mouse line. Overexpression of the Cdk5 activator, p25, in mouse thyroid C cells leads to the development of MTC, while preventing p25 overexpression arrests tumor growth. These findings demonstrate clearly the importance of Cdk5 activity for MTC progression. This was at first intriguing because Cdk5 is better known as a neuronal protein. Indeed, Cdk5 and its activators are expressed at high levels in brain compared to other tissues. Cdk5 regulates brain development and function and deregulation of Cdk5 activity by binding to its cleaved activator, p25, has been linked to neurodegenerative diseases such as Alzheimer's disease. Considering that neuroendocrine cells and neurones have a common ontological origin (the neural crest) and that they share similar properties (e.g. calcium-dependent release of neurotransmitters or hormones), this study raises the possibility that common signalling pathways may underlie cancer and neurodegenerative diseases.

**Figure 1:**
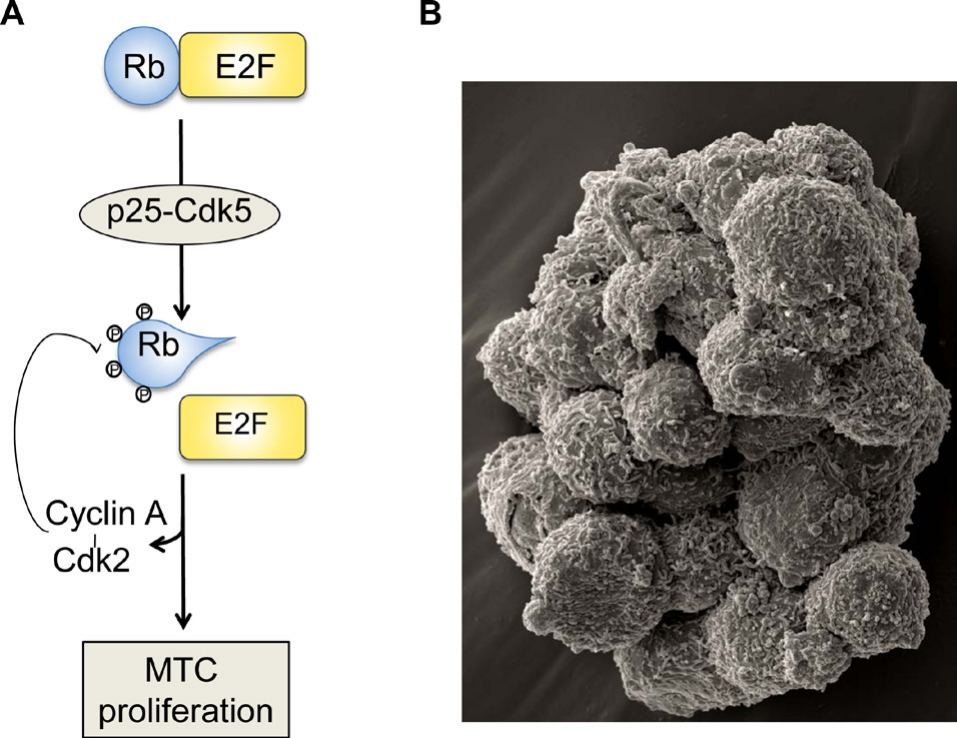
Cdk5 contributes to neuroendocrine thyroid cancer tumorigenesis A, a schematic model showing the proposed mechanism by which Cdk5 mediates MTC proliferation; B, a scanning electron micrograph of cultured mouse thyroid C cells that were derived from a mouse MTC; the cells grow in suspension, as floating clusters. Assistance for SEM was provided by Anza Darehshouri and Robyn Leidel of the UT Southwestern Electron Microscopy Core Facility for assistance with the Zeiss Sigma VP SEM, on loan to UTSW from Carl Zeiss Microscopy.

Mechanistically we found that Cdk5 regulates the phosphorylation state of the tumor suppressor, retinoblastoma protein (Rb), and the subsequent expression of Cdk2 and cyclin A (Figure 1A). It is well accepted that cell cycle progression from G1 to S phase is dependent on the initial phosphorylation of Rb by cyclin D-Cdk4/6 and on the maintenance of Rb phosphorylated state by cyclinA/E-Cdk2 complexes [5]. Our findings suggest that this may not be true in neuroendocrine cells and that Cdk5 mediates the initial Rb phosphorylation instead of Cdk4/6. This may explain why certain cancer cell lines are resistant to Cdk4/6 inhibitors. To verify this hypothesis, it will be important to determine whether MTC cell lines are resistant to Cdk4/6 inhibitors and conversely whether Cdk4/6 resistant cells are sensitive to Cdk5 inhibitors.

In this study, we report a novel animal model for MTC based on a tetracycline-controlled transactivator system (tTA) in which we can induce or arrest MTC development by activating or repressing p25 overexpression in the mouse thyroid C cells (Figure 1B). We showed that Cdk5 activity was highly elevated in growing tumors compared to arrested tumors. Hence, we can generate and collect identical MTC tissues that differ only by their Cdk5 activation levels. By comparing the molecular content of arrested and growing tumors, we can gain a deep insight on the Cdk5-mediated mechanisms contributing to MTC progression. We used this comparative approach to identify Rb, Cdk2 and cyclin A as downstream effectors of Cdk5. Thus our animal model is a useful tool to identify new mechanisms underlying MTC tumorigenesis. In addition, this clinically relevant model of MTC can be used for the preclinical testing of new therapies.

In conclusion, our study unravels a major role for Cdk5 in neuroendocrine thyroid cancer and suggests that targeting Cdk5 or downstream effectors is a valid strategy to cure MTC. Furthermore our animal model provides new routes to discover molecular pathways important for MTC and test potential therapies. Our findings represent an important advance in a field in which molecular tools are limited and the discovery of new putative drug targets difficult.
